# Gravitational body forces focus North American intraplate earthquakes

**DOI:** 10.1038/ncomms14314

**Published:** 2017-02-17

**Authors:** Will Levandowski, Mark Zellman, Rich Briggs

**Affiliations:** 1 United States Geological Survey, Geologic Hazards Science Center, P.O. Box 25046, DFC MS966, Denver, Colorado 80225, USA; 2 Fugro Consultants Inc. 1726 Cole Boulevard, Suite 230, Lakewood, Colorado 80401, USA

## Abstract

Earthquakes far from tectonic plate boundaries generally exploit ancient faults, but not all intraplate faults are equally active. The North American Great Plains exemplify such intraplate earthquake localization, with both natural and induced seismicity generally clustered in discrete zones. Here we use seismic velocity, gravity and topography to generate a 3D lithospheric density model of the region; subsequent finite-element modelling shows that seismicity focuses in regions of high-gravity-derived deviatoric stress. Furthermore, predicted principal stress directions generally align with those observed independently in earthquake moment tensors and borehole breakouts. Body forces therefore appear to control the state of stress and thus the location and style of intraplate earthquakes in the central United States with no influence from mantle convection or crustal weakness necessary. These results show that mapping where gravitational body forces encourage seismicity is crucial to understanding and appraising intraplate seismic hazard.

## Abstract (web summary)

Intraplate earthquakes occur far from tectonic plate boundaries and so it is vital to understand how and where they may happen. Here, Levandowski *et al*. create a 3D density map of the North America Great Plains showing that gravitational forces play a controlling role in intraplate earthquake locations.

If the interaction between plates at their distant boundary were the only source of deviatoric stress in continental interiors, then the intraplate stress field would be broadly uniform, and all suitably oriented faults would have equal chance of experiencing an earthquake. Yet many ancient fault zones remain seismically quiet, and even high-rate wastewater injection does not everywhere induce seismicity^1^. Intraplate earthquakes tend to cluster in discrete zones, many of which are ancient tectonic features that have reactivated multiple times throughout geologic history^2^,^3^,^4^,^5^. Because tectonism may cause crustal thickening or thinning, metamorphism, or other alterations to the density of the lithosphere, such multiply deformed zones may host lateral lithostatic pressure disequilibria that now serve as localized sources of stress^6^,^7^,^8^,^9^,^10^ capable of encouraging or suppressing future slip on associated faults^9^.

The Great Plains of the central United States host two zones of recurrent strain localization and modern seismicity: a pair of NE-striking Proterozoic terrane boundaries, the Yavapai–Mazatzal suture zone (YMS) in SE Colorado and the Cheyenne Belt (CB) in SE Wyoming and the Nebraska Panhandle (Fig. 1). In the Paleoproterozoic, North America grew southward by accretion of the Yavapai terrane on the CB and then the Mazatzal block on the subparallel YMS^11^. Paleozoic compression reactivated faults in each suture zone, impounding the Denver basin and the NW end of the Anadarko basin^12^,^13^. The Cenozoic Laramide orogeny created 3 km of structural relief in the CB^14^ (exhuming Archean rocks) and reactivated the YMS^15^, where units as old as Permian are now locally exposed.

The Miocene Ogallala alluvium has tilted up to the west on the eastern part of the CB^16^, and its base bows upward several hundred metres across the YMS^4^,^17^. Although erosional unloading due to post-Miocene climatic changes^18^ is a plausible source of such rock uplift, the Cheraw Fault within the YMS has generated 3M∼7 down-to-NW extensional surface-rupturing earthquakes since 25 ka (ref. 19) and manifests as a 46 km long NE-trending fault scarp. Similarly, well data suggest up to 200 m of vertical throw on the Wheatland/Whalen fault system within the CB since the mid-Miocene^20^. Therefore, tectonic processes may contribute to post-Miocene rock uplift on the YMS and CB.

Both Proterozoic boundaries are foci of modern seismicity (Fig. 1). Earthquakes in SE Wyoming and NW Nebraska follow the north flank of the CB, and a trend of earthquakes on the YMS collinear with the Cheraw Fault extends 400 km northeastward from the Raton basin at the Rocky Mountain front onto the Great Plains. Earthquake moment tensors and borehole breakouts^21^ (earthquakes.usgs.gov/search) reveal that the principal stress directions are different along the YMS and CB than in their surroundings. Induced seismicity in the Denver and Raton basins accommodates down-to-E normal faulting, and earthquakes both east and west of the CB reveal NE–SW extension. Therefore the NW–SE extension on the Cheraw and Wheatland/Whalen faults represents 45–90° rotations of the regional stress field. Because seismicity occurs both near and at great distance from the Arkansas and North Platte thalwegs, focused erosion due to post-Miocene climatic changes cannot explain these patterns. And although uniform erosion would very slightly increase the Coulomb stress on buried faults, bringing them closer to failure, it cannot account for anomalous principal stress directions.

By contrast, local augmentation of tension normal to the YMS and CB could both focus and reorient seismicity^7^,^8^. The net stress tensor is the sum of those due to edge-normal, edge-shear, basal-normal, basal-shear and gravitational stresses. Yet, variations in stress applied at plate boundaries, some 2,000 km distant, are a poor explanation for such small-scale features. In addition, modelled eastward mantle flow^22^ associated with the Rio Grande Rift and/or Farallon slab would create E–W compression in the overlying lithosphere, and modelled downwelling where the eastward-flowing asthenosphere encounters the thicker lithosphere of the Plains (in the vicinity of the YMS^22^) would produce downward basal-normal stress and thus favour contraction in the overlying material. Since these basal forces oppose NW–SE extension (and also the broadly E–W extension observed throughout the western Plains; Fig. 1), gravitational body forces seem the most plausible source of local stress augmentation and reorientation.

We test the hypothesis that gravitational body forces promote failure of faults in and near these sutures. To do so, we construct 1,000 3D lithospheric density models of the Southern Rockies and Plains jointly from seismic velocity, gravity and topography (see Methods and Supplementary Figs 1–10). We then use both analytical and numerical methods to calculate the body forces attendant to these 3D density structures.

## Results

### Anomalous lower crust in Proterozoic suture zones

**Figure 1:**
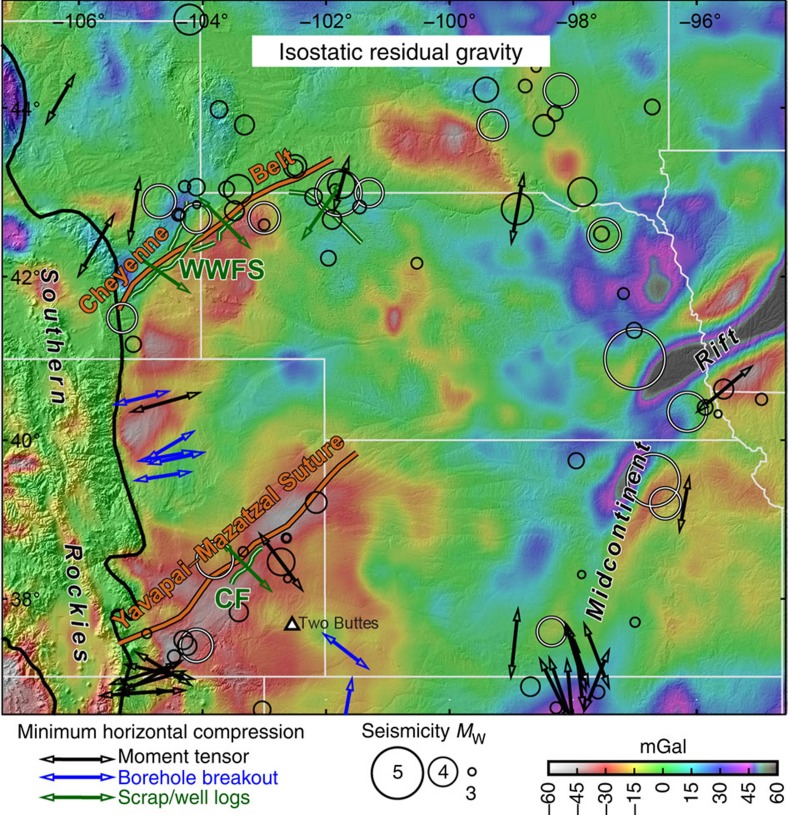
Overview map of tectonic and seismotectonic features. Natural earthquakes are shown as circles; events in the Rockies are omitted from this figure for clarity. The Holocene-active Cheraw Fault (CF) lies within the Proterozoic Yavapai–Mazatzal suture zone and is collinear with a NE–SW trend of epicenters. Similarly, the post-Miocene-active Wheatland–Whalen fault system (WWFS) is within the Cheyenne Belt—the suture between the Archean Wyoming craton and the Yavapai block—and a relatively seismically active part of the Great Plains. Most the remaining seismicity occurs in the vicinity of the Proterozoic Midcontinent Rift. Arrows show minimum horizontal compression directions from geologic, geophysical and geotechnical indicators: black, earthquake moment tensors; blue, borehole breakouts; green, fault scarps or well logs.

**Figure 2:**
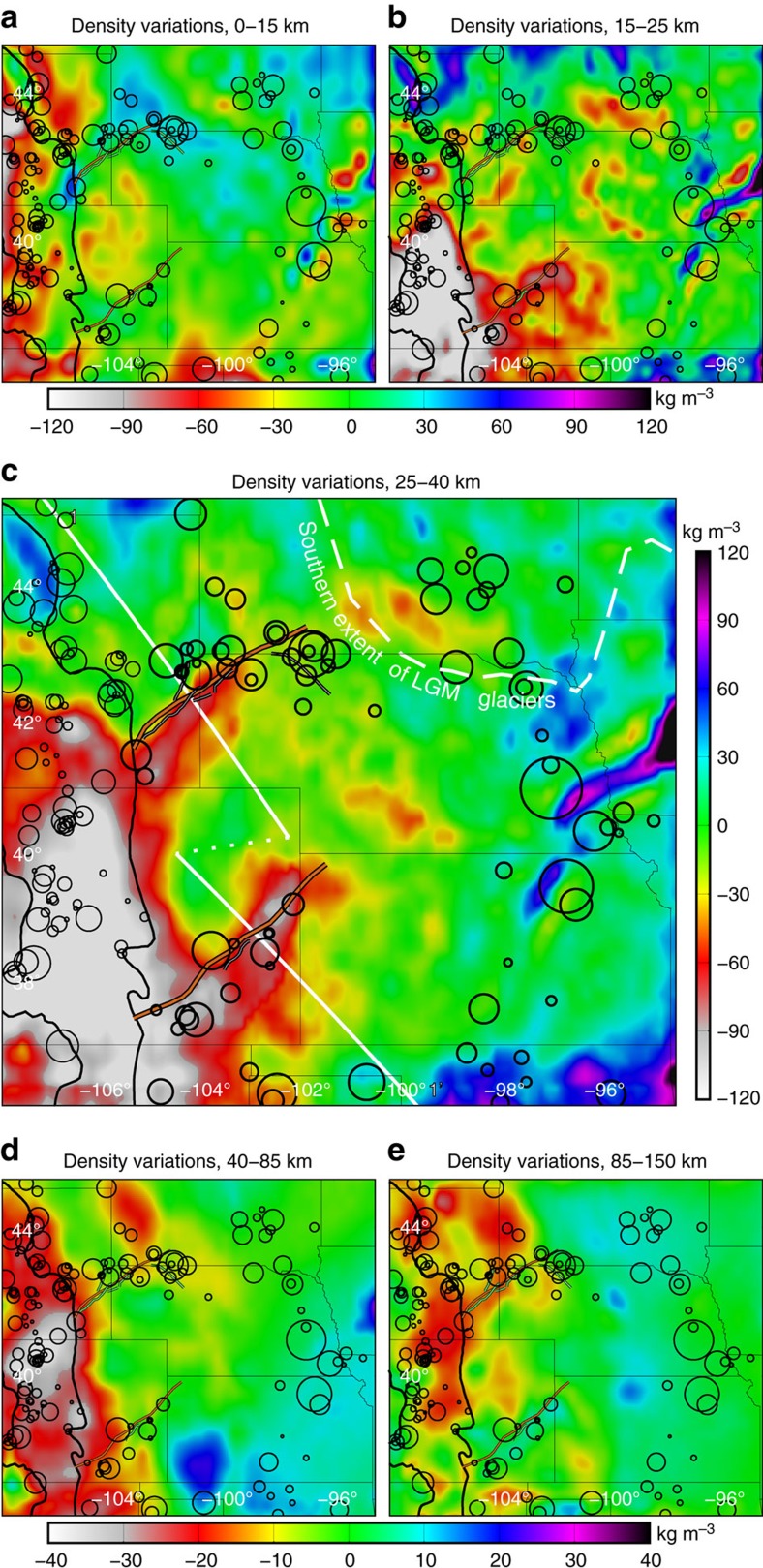
Depth-slices through the mean density model. The mean is removed from each depth range to facilitate comparison. Note buoyant lower crust beneath the Proterozoic sutures and dense material beneath the Midcontinent Rift in **c**. Because of the narrower range of densities in the mantle, the scale of **d**,**e** is narrowed relative to **a**–**c**. Seismicity is as in Fig. 1, but with events in the Rockies included.

**Figure 3:**
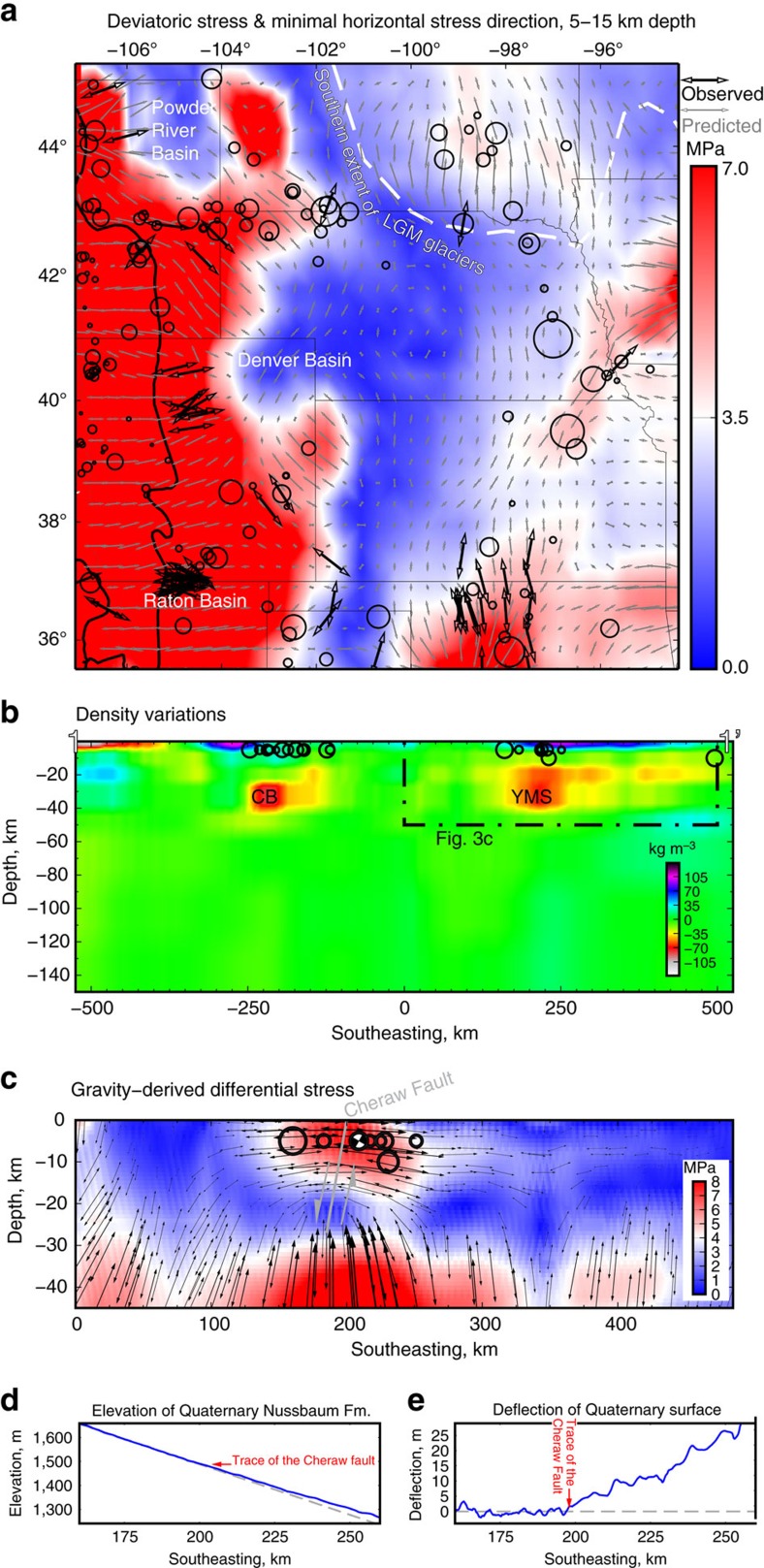
Stress and strain due to gravitational body forces. (**a**) Estimates of deviatoric stress in the seismogenic crust from 3D finite-element modelling. The predicted horizontal tension directions are shown in grey, with the observed stress indicators in black for comparison. There is generally good agreement between predicted and independently observed tension directions. As in previous figures, black circles denote natural seismicity. Most earthquakes occur in areas of elevated stress. (**b**) Lateral density variations along fence diagram 1–1′. Location shown in Fig. 2c. All earthquakes within 150 km of the plane are plotted. All but one falls within the YMS or CB. (**c**) 2D finite-element model of gravity-derived stress due to the structure in **b**, with results for the vicinity of the Cheraw Fault shown. Arrows denote maximal tension direction. The one focal mechanism available is shown. Buoyant lower crust produces tension in the overlying material, consistent with suture-normal extension. (**d**) Topographic profile along a continuous surface of the ∼1.4 Ma Nussbaum alluvium where it crosses onto the flank of the YMS. Horizontal scale is as in **b**,**c**. Blue line: surface elevation. Grey line: the average slope NW of the trace of the Cheraw Fault. (**e**) Surface deflection, or the blue line in **d** minus the grey line. The decreased slope on the YMS is consistent with ∼3 cm per kyr of uplift, as proposed by ref. 17. For comparison, average erosion rates on abandoned terraces are ∼10–15 cm per kyr in the region^18^.

The lower crust beneath each the YMS and CB is anomalous: as much as 100 kg m^−3^ less dense than beneath the surrounding Plains (Figs 2c and 3b). Independent of our density modelling, high seismic velocity in the upper crust and thin or absent sedimentary cover—with local exposures of Permian (YMS) and Archean (CB) rocks—coincident with low gravity (for example, Fig. 1) suggest relatively dense shallow crust underlain by buoyant material.

Quartz-rich^23^, hot^9^ and hydrous^24^ lower crust accord with the buoyant bodies that we image in the Proterozoic suture zones. Yet sparse volcanism that furthermore is mafic in the Raton-Clayton field^4^ and Two Buttes^25^ near the YMS (Fig. 1) likely precludes large felsic intrusions into the lower crust, and the −100 kg m^−3^ density anomaly would require a thermal anomaly of +1,100 °C (for a coefficient of thermal expansion of 2.5 × 10^−5^ per °C and a reference density of 2,800 kg m^−3^), and therefore a temperature implausibly higher than the solidus.

Hydrous lower crust beneath the YMS and CB provides the best explanation for the observed density anomalies. Garnet, pyroxene and feldspar may retrogress to chlorite, albite and calcite in the presence of water, reducing seismic velocity and dramatically decreasing density^24^. Lithospheric-scale damage zones inherited from prior tectonism are likely to be more efficient conduits than surrounding lithosphere^3^,^26^,^27^, so flux from dewatering of subducted slabs (either Farallon^2^,^24^,^28^ and/or earlier^26^,^27^) into the overlying lithosphere should be greater along ancient sutures.

Geochemical data and xenoliths verify the existence of hydrated lithosphere beneath at least the YMS. The 37 Ma Two Buttes minette, 100 km SE of the Cheraw fault, sourced in hydrous mantle lithosphere^25^. Crustal xenoliths found near the suture on the Colorado Plateau^26^ record pervasive hydration-induced retrogression of garnet-bearing assemblages; Th-Pb ages of secondary monazite in these products are dominantly (>60%) 60–90 Ma (ref. 29), contemporaneous with the putative arrival of the flattening Farallon slab.

### Stress due to density variations

If lower crustal dedensification did occur during the Laramide orogeny, then it coincided with the change from contraction in the YMS and CB in the Proterozoic, Paleozoic and early Tertiary to Miocene and modern extension. We derive a simple, proof-of-concept 2D analytical model to test whether buoyant lower crust would in fact promote extension in the overlying material. This analytical solution shows that a −100 kg m^−3^ density anomaly between 20 and 40 km depth creates 10 MPa of horizontal tension in the material above it (Supplementary Fig. 11), consistent with suture-normal NW–SE extension on the Cheraw and Wheatland/Whalen faults.

This model also predicts ongoing rock uplift along the YMS and CB, and we find geomorphic evidence for up to 30 m of Quaternary uplift on a continuous surface of the ∼1.4 Ma Nussbaum alluvium near the Cheraw fault (Fig. 3d,e). Indeed, 10 MPa of deviatoric stress acting on a Newtonian material of 10^23^ Pa·s viscosity for example, ref. 30 induces 3 × 10^−9^ strain per year. Over 1 Myr ago, a body 200 km wide by 20 km thick would shorten 600 m. To conserve cross sectional area, the body would extend vertically, but only by 26 m. These values, arbitrary and approximate as they are, do closely resemble our observations (Fig. 3d,e). Extrapolating this line of reasoning predicts some 250 m of rock uplift since the deposition of the Miocene Ogallala formation, similar to the amount of tectonic uplift proposed^17^.

### 3D model of gravity-derived stress on the Great Plains

Although the analytical solution shows unambiguously that buoyant lower crust should generate horizontal tension in the material above it, we seek to estimate the direction and magnitude of modern principal stress in three dimensions throughout the seismogenic crust in our study area. Specifically, we wish to investigate the extent to which predicted stress magnitudes correlate with rates of natural seismicity. A complementary independent test of the role of body forces is available by comparing the predicted principal stress directions with those that are observed in earthquake moment tensors and borehole breakouts.

To do so, we use our density model as input to 3D finite-element simulations and test whether modelled stress magnitudes and directions explain the spatial distribution of earthquakes and observed principal stress directions. Consistent with the simplified analytical solution, these models again predict up to 10 MPa of NW–SE deviatoric tension on the sutures, which is anomalous in both direction and magnitude relative to the rest of the western Plains (Fig. 3a,c).

Our analysis also highlights regions other than the YMS and CB where body forces create large deviatoric stress (Fig. 3a). The most prominent feature of this model is the uniformly high stress modelled in the southern Rockies. In accordance with this prediction, rates of natural seismicity in the Rockies are generally higher than on the Plains (Fig. 3a). And aside from the YMS and CB, the other main focus of natural seismicity on the Plains surrounds the Midcontinent Rift (for example, Fig. 1). There, anomalously dense middle and lower crust—some 100 kg m^−3^ denser than its surroundings (Fig. 2b,c)—elevates gravity-derived deviatoric stress at seismogenic depths relative to the rest of the Plains (Fig. 3a). The coincidence of the three foci of earthquakes on the Plains with zones of elevated gravitational stress supports the thesis that body forces are an important factor in natural intraplate seismicity. Indeed, most of the natural seismicity on the Plains—especially outside of the footprint of Wisconsonian glaciation—has occurred in areas of elevated stress.

In addition, although wastewater injection-rate locally controls the spatial distribution of induced seismicity, a high proportion of disposal wells in these three regions are spatiotemporally associated with earthquakes^1^ (in the Raton basin, on the margins of the Powder River basin but not within it, and in southern Kansas, specifically). By contrast, in regions with abundant injection wells but without anomalous densities—the Denver basin, the interior of the Powder River basin, and much of central and western Kansas—induced seismicity is generally rare^1^. Just as body forces appear to control strain on the Great Plains, *in situ* stress may also predispose certain locations to induced seismicity.

Moreover, body forces alone are capable of reproducing the principal stress directions observed in moment tensors across the region. Roughly, E–W normal faulting is indeed predicted in the Denver and Raton basins and on the western side of the Powder River basin. Over distances of ∼200 km, tension rotates to NW–SE on the YMS and CB. Farther east, approximately north-south extension is predicted—and observed coseismically—across a broad region from central Nebraska to central Oklahoma. Finally, the same dense crust that elevates deviatoric stress near the Midcontinent Rift also controls stress orientations: Moment tensors record rift-parallel minimum stress along the rift-flanks, reflecting the rift-normal compressional influence of the adjacent, dense intrusive rocks. We thus conclude that gravitational body forces appear to exert the primary control on the magnitude and direction of principal stress on the Great Plains and therefore on the location and style of intraplate seismicity in the region.

### The role of far-field stress

This conclusion contrasts with the common conception that the stress field in North America is dominated by edge stresses—specifically ENE–WSW horizontal compression from Pacific-North American transpression and Mid-Atlantic ridge push for example refs 30, 31—or by basal stress due to mantle flow, either from Farallon subduction^32^,^33^ or small-scale convection^22^,^34^,^35^. Stress due to density variations would be superimposed upon this regional stress (although regional approximately E–W compression would be at odds with the broadly E–W extension observed from the westernmost Great Plains through the Basin and Range), so the tensor sum of this background stress field with our estimate of gravity-derived stress allows us to constrain the plausible magnitude and best-fitting direction of regional stress^8^,^36^,^37^,^38^.

Surprisingly, we find that the best correspondence between predicted and observed horizontal extension directions is achieved by gravity-derived stress alone, though 1–2 MPa of NE–SW to E–W far-field compression does not substantially degrade the results (Supplementary Fig. 13b,h,j). Greater magnitudes and other orientations of regional stress worsen the fit substantially (Supplementary Fig. 12a,c–g,i). This finding suggests that the relative contribution of density variations to long-term fault loading matches or exceeds that of edge-stress or basal shear.

## Discussion

Earthquakes, both natural and induced, on the Great Plains of intraplate North America occur in discrete zones. We have shown that these foci of seismicity occur where lithospheric density structure elevates deviatoric stress. Beyond this spatial coincidence, the principal stress directions predicted by finite-element models of gravitational body forces closely reproduce the stress directions observed independently in earthquake moment tensors. Therefore, lithospheric density structure appears to play a large—and to some extent deterministic—role in controlling the local, long-term strain-rate tensor across a large portion of North America.

This observation opens the possibility of improving time-independent seismic hazard models in continental interiors. Currently, the source models used in such assessments are inherently retrospective insofar as they are based on catalogues of instrumentally recorded, felt and geologically documented earthquakes from the past^39^. If indeed the long-term strain- and therefore earthquake-rate in intraplate settings is governed by the lithospheric-scale deviatoric stress^36^, then the long-term seismic moment tensor should be proportional to the local stress tensor. Quantifying whether these local variations in stress promote or inhibit slip on known or hypothesized fault planes would allow for consideration of seismic sources for which the Quaternary slip-record is incomplete. Consequently, understanding local stress and its fault zone-scale variations as we have attempted here could provide an avenue toward prospective (albeit still time independent) seismic hazard models in intraplate settings.

## Methods

### Density modelling

The density model is derived following ref. 40. Hundreds of plausible joint seismic velocity-crustal thickness models—derived jointly from short-period ambient noise surface wave dispersion, long-period (to 80 s) ballistic surface wave dispersion, and receiver functions—at each of ∼1,000 Transportable Array stations^41^ are used to create starting density structures in three dimensions on a 20 × 20 km grid. Following Levandowski *et al*.^42^ we use seismic velocity to estimate lithospheric density (Supplementary Fig. 2).

In the crust, we estimate the contribution of thermal variations to reported velocities and scale remaining velocity variations to density using isothermal empirical regressions of density onto velocity for a variety of rock types. On the basis of empirical data from refs 43, 44, we derive the following regression:


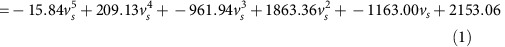


Then, the temperature variations that we have previously estimated are scaled to density variations; these sum with the inferred compositionally derived densities.

In the mantle, we use a scaling of velocity to density that accounts for temperature variations, seismic anelasticity, and melt production but that does not consider compositional variations. We calculate the relationships between velocity and temperature and between density and temperature for six common upper mantle minerals: Fo_90_, Fo_92_, orthoenstatite, ferrosillite, garnet and spinel. To do so, we use (1) published estimates of the shear and bulk moduli and their pressure- and temperature-derivatives^45^,^46^,^47^,^48^, (2) temperature-dependent thermal expansivities^48^ and (3) a temperature-, pressure- and seismic-period-dependent calculation of the dynamic compliance, or the Laplace transform of the creep function using scripts provided by U. Faul^49^, to account for anelasticity. Since the velocity models we use are based on surface wave dispersion, which senses increasing depths with increasing periods (from ∼50 km at 30 s to 150 km at 80 s, the longest period used by Shen *et al*.^41^), we must employ a depth (pseudoperiod) dependence of the velocity-to-density scaling. Fitting the velocity–temperature and density–temperature curves for bulk pyroxenitic composition (30% Fo_90_+30% Fo_92_+25% OPX+10% CPX+2.5% Gt+2.5% Sp) at a variety of depths—or the attendant pressure and dominant surface wave period—yields a relationship between velocity and density deviations from the assumed solidus (*v*_*s*_=4.5 km s^−1^; *ρ*=3,200 kg m^−3^) at a depth *z*, a velocity perturbation (*Δv*_*s*_ quantified as per cent of 4.5 km s^−1^) scales to a density perturbation as:


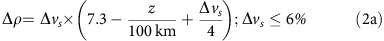



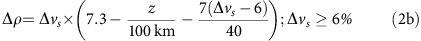


The variations in this scaling among realistic compositions of the upper mantle are minor. The density change of depleted (Mg-, OPX- and Ol-rich) material that corresponds to a unit velocity change is 5% greater. Fertile (Fe-, Gt-/Sp- and CPX-rich) material is 5% less sensitive. To account for melt-production, velocities below the assumed solidus (here 4.5 km s^−1^) correspond identically to a density of 3,200 kg m^−3^, since melt-production does not substantially alter density.

Although the approach outlined above could be tailored to different mantle compositions, equations (2a-2b), 3 only account for the differing thermal properties and moduli of minerals, not their densities nor velocities at some reference state. Specifically, the compositional trends associated with melt-depletion strongly lower density but slightly increase *v*_*s*_. Quantified in terms of an associated unit increase in olivine Mg#, melt-depletion (dominantly the loss of garnet/spinel) lowers density ∼12 kg m^−3^; conversely, *v*_*s*_ increases 0.25% (ref. 51). The density of this material (by equations (2a-2b), 3) would be overestimated by some 14 kg m^−3^. Since our scaling of mantle velocity to density is relatively insensitive to compositional variations, the differences between fertile and depleted mantle manifest as topographic and, to a lesser degree, gravity residuals.

Such seismically derived estimates for example ref. 42 reproduce most of the gravity and flexurally modulated topography variations in the Great Plains and Southern Rockies (to within 150 m and 15 mGal, L1 norm; Supplementary Fig. 1a–f). Nevertheless, features finer than the lateral resolution of seismic velocity models and some regional-scale variations are unexplained (Supplementary Fig. 1e,f). To produce a higher spatial-resolution, more precise density model, we use the random-walk Monte Carlo algorithm of ref. 40 to iteratively refine the seismically derived estimates (Supplementary Figs 2 and 3) until gravity and flexural topography are explained to within 5 mGal and 50 m across the region (Supplementary Fig. 1g,h). These adjustments are generally small compared with lateral variations inferred from velocity and compared with the uncertainty in velocity or in velocity–density scaling (Supplementary Figs 4–7). We conduct 1,000 simulations, each beginning by selecting at random one of the hundreds of velocity models provided at each TA stations. As such, our approach is more data-driven than one using a single (for example, smoothest, best-fitting or otherwise chosen *a priori*) velocity model: As long as a given velocity model can acceptably reproduce the seismic data, it is considered as a starting point. The results we discuss are the mean across those 1,000 simulations. Uncertainties and biases are discussed further in Supplementary Methods.

Gravity calculations approximate each cell as a rectangular prism and compute the vertical component of gravitational attraction at each surface node due to a unit density in each cell. The same kernel can then be used in the Monte Carlo simulations. The 20 × 20 km grid is divided into 16 layers: surface to sea level, 12 5-km layers from seal level to 60 km and then 3 30-km layers from 60 to 150 km. We interpolate the initial density model horizontally (by Delaunay triangulation), and each cell has a uniform density. In the initial calculation, we remove the mean from each layer in order to mitigate edge effects (except the surface layer, which is treated similarly to a Bouguer slab, though with laterally variable density estimated from seismic velocity). This estimate (for example, Supplementary Fig. 1d) is then compared with observed free air gravity^51^ (for example, Supplementary Fig. 1b).

This initial density model predicts local isostatic topography, *E* (ref. 52):


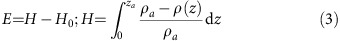


*H*_*0*_ is a correction term of 2.4 km to achieve isostatic equilibrium with an asthenospheric column (via mid-ocean ridges). The mantle below the base of the seismic models at 150 km depth (*z*_*a*_) is assumed to be laterally uniform, with a density (*ρ*_*a*_) of 3,200 kg m^−3^. We account for the flexural strength of the lithosphere by convolving *E* with the flexural filter of the lithosphere, *F*, resulting in the smoothed surface elevation field, *ɛ*_predicted_ (Fig. S1c), predicted from the density model:


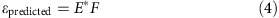


*F* is a system of zero-order Kelvin-Bessel functions; as given by ref. 53; the deflection *w*(*r*) of a plate with flexural parameter *α* at radius *r* from the center of a cylindrical load of height *h*, radius *R*_*d*_, and density anomaly *Δρ* is:


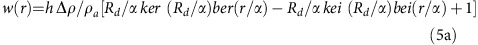


within the load (that is, for *r<R*_d_), and


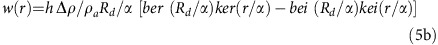


outside of the load (that is, *r>=R*_*d*_).

Here ber, bei, ker, kei, ber', bei', ker' and kei' are zero-order Kelvin–Bessel functions and their derivatives. We use the elastic thickness estimates of ref. 54 to calculate 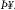. *R*_*d*_ is calculated to preserve the ground-surface area of each cell (that is,, 28 km for a 20 × 20 km cell).

Much as the flexural strength of the lithosphere modulates the topographic expression of subsurface loads, topography can be partially supported by the flexural strength of the lithosphere. To this end, the observed elevation field is convolved with *F* (equations (4) and, (5a) and ) to produce a smooth elevation field *ɛ*_observed_ (Supplementary Fig. 1a). The topographic residual (Supplementary Fig. 1e) is the difference between *ɛ*_predicted_ and *ɛ*_observed_.

### Resolution tests

High seismic velocity in the upper crust and thin or absent sedimentary cover—with local exposures of Permian (YMS) and Archean (CB) rocks—suggest relatively dense shallow crust (for example, Supplementary Fig. 2a). Nevertheless, to determine whether the low-density material beneath the CB and YMS could possibly be an artifact of our modelling, we test the ability of the density refinement algorithm to recover *a priori* density anomalies (that is,, mimicking density variations not manifest in seismic velocity). We first calculate the gravitational and topographic effect of the input bodies and then invert these signals.

Two tests are of interest to the present study: could the low-density modelled in the lower crust simply be smeared downward from the shallow crust? If the lower crust is indeed anomalously buoyant, how faithfully does the Monte Carlo algorithm recover the magnitude of this anomaly? Results (Supplementary Figs 9 and 10) are from 100 simulations (rather than the 1000 used for real data).

In each case, the anomaly is smeared somewhat vertically (Supplementary Figs 9 and 10). A −100 kg m^−3^ density anomaly confined to the upper 15 km is modelled as −70 kg m^−3^ from 0 to 15 km and −20 to −30 kg m^−3^ from 15 to 40 km. Nevertheless, the −100 kg m^−3^ density anomaly in the lower crust modelled from real data cannot plausibly be only an artifact of shallower structure (especially considering the known geology and shallow structure). Similarly, an input −100 kg m^−3^ anomaly from 15 to 40 km is modelled as −25 kg m^−3^ from 0 to 15 km and −70 kg m^−3^ from 15 to 40 km. Therefore, if the density anomaly is confined to the lower crust, the magnitude discussed in the main text is likely a conservative estimate.

In summary, the vertical resolution of our density refinement algorithm is imperfect, but it is not plausible that the buoyant lower crust imaged beneath the YMS and CB is an artifact of shallower crustal structure. In addition, since the two suture zones are structural highs—especially compared to the Powder River basin northwest of the CB, the Denver-Julesburg basin between them, and the Anadarko basin (where sedimentary units may extend as deep as 15 km for example, ref. 56)—there is geologic evidence to suggest that the upper crust beneath the suture zones is indeed denser than beneath adjacent regions, as we image independently from geophysical constraints.

### Uncertainties of density

There are three primary sources of uncertainty in our density modelling: the natural variability of seismic models capable of reproducing surface wave dispersion curves and receiver functions at the ∼70 km spacing of Transportable Array stations, the epistemic uncertainty of the true density of material given knowledge of its seismic velocity, and the fact that gravity is a non-unique function of 3D density structure.

The hundreds of independent seismic velocity models at each station produce a prior distribution of density estimates in 3D (Supplementary Figs 4 and 5). A typical uncertainty or the range that subsumes 95% of all velocity models at a given depth beneath one station, for the velocity models is ∼0.15 km s^−1^ through most of the range 0–150 km. Uncertainties are somewhat larger in the uppermost 1–3 km (±0.5 km s^−1^; due to a loss of sensitivity below the microseismic peak) and lower crust (±0.4 km s^−1^; due to tradeoffs between lowermost crustal velocity and the magnitude of the velocity jump across the Moho). The suite of velocity models thus produces a prior distribution of density estimates with aleatory uncertainties of ∼50 kg m^−3^ (the mean across all stations) in the mid-crust and mantle, ∼150 kg m^−3^ in the lower crust and >200 kg m^−3^ in the few kilometre nearest the earth's surface (Supplementary Figs 4 and 5).

The uncertainty attendant to the seismic velocity models is, nevertheless, far too conservative an estimate of the actual uncertainty of the density structure given only seismic velocity models, most especially because density cannot be precisely known from seismic velocity alone. Crustal lithology, metasomatism of the crust and/or mantle lithosphere, mantle melt-depletion and many other factors affect density and velocity in ways not captured by equations (4), (5a) and . And other factors such as seismic anisotropy impact velocity but not density. For example, we have previously shown Fig. 2a of ref. 40 that any reasonable regression through empirical velocity–density data from crustal liithologies leaves behind residuals of up to ±150 kg m^−3^; generally, mafic rocks are denser than predicted, and felsic rocks are typically less dense. Similarly, the hydration gradient from pure peridotite to serpentinite produces 0.59% decrease of S-velocity per 1% serpentinite but −8.3 kg m^−3^ per 1% serpentinite^56^; thus, each 1% decrease in S-velocity from hydration is accompanied by 14 kg m^−3^ density loss, approximately twice the change as inferred from equations (5a) and (5b). Similar steep drops in density relative to velocity due to crustal hydration are shown by Jones *et al*.^24^ their Fig. 3a for crustal xenoliths from the western Great Plains. Myriad other trends affect the velocity and density of rocks differently, and an exhaustive list is unnecessary. The most prominent, however, is melt-depletion of the mantle lithosphere. Loss of iron, garnet and volatiles and increase in modal abundance of olivine and magnesium causes a decrease in density but even a slight increase of S-velocity^50^,^57^; thus the scaling of velocity to density would greatly overestimate the density of melt-depleted regions. Finally, seismic anisotropy, aligned cracks in the shallow crust, and a great many other factors can affect seismic velocity but not density.

As such, there is no reason to take the estimate of density garnered from seismic velocity as more than a starting point. A 3D density structure must, however, reproduce gravity and topography to be viable. In our parameterization, the starting model should accurately estimate the densities of intermediate crustal rocks, and most crustal and upper mantle density variations due to temperature, and it should do so at the ∼100 km horizontal resolution of the seismic velocity models. Consequently, the departures from this initial estimate that are adopted during the Monte Carlo inversion plausibly represent anomalously mafic or felsic crustal bodies of a few 10s of km of lateral dimension, hydrous crust or mantle lithosphere, melt-depleted mantle lithosphere, or simply features that are smaller than the resolution of the seismic velocity models (note short-wavelength gravity residuals in Supplementary Fig. 1f and the short-wavelength adjustments required in Supplementary Fig. 3).

Although gravity and topography are both non-unique functions of 3D density structure, when taken together—since the vertical component of the gravity field is sensitive to 3D mass distribution and topography is sensitive to 2-D buoyancy—the two actually do prescribe a unique density structure. In practice, however, an infinite number of 3D density structures could reproduce both fields to nearly arbitrary precision. For this reason, we conduct 1,000 simulations, explicitly embracing non-uniqueness.

We have mentioned the variability of the velocity and thus the prior distribution (that estimated from the velocity models using equations 7 and 8) of density at any given point. In practice, however, the smoothness of surface wave dispersion kernels with depth—in other words, the vertical covariance in the individual 1D velocity models—implies that a more meaningful quantity to track is the uncertainty of the average velocity over some depth range, or for our purposes the variability of, say, lower crustal density across the prior distribution. Similarly, gravity has essentially no sensitivity to the density in an arbitrarily small volume. Gravity is sensitive to the volume-integral of density, the total mass within a certain volume: even a small body with a physical negative density is not precluded by the gravity data, the body would simply need be surrounded by appropriately denser material. The same is true for topography, except that it depends on 1D depth-integrals of density rather than volume-integrals of density. So again, the meaningful quantity is not the density or the uncertainty at a given point but rather in a volume/depth range of interest.

We quantify the uncertainty of the initial and final models in three dimensions (Supplementary Figs 4–7). At all depths, there is a strong correlation between the initial and final uncertainty (Supplementary Fig. 7). Such a result is to be expected, since the seismic velocity model broadly reproduces observed gravity and topography (for example, Supplementary Fig. 1e,f); the dominant structures revealed by the seismic images are preserved, and adjustments are generally small in magnitude and/or lateral extent. Said differently, the lateral variations in the initial density estimates (those inferred from velocity models) at any given depth are much greater in magnitude than the adjustments to them (compare Supplementary Figs 2 and 3). The best correlation between the uncertainty of the initial models and of the final models—as well as the highest uncertainties in both—is found near regional Moho depths, approximately 30–60 km. In this depth range, typical 2σ uncertainties are ∼35 kg m^−3^. Correlation between initial and final uncertainty is nearly as good in the mantle, though because the total range of densities is inferred to be smaller than in the crust, uncertainties across the prior and posterior distributions are much smaller: 10 kg m^−3^ on average.

The 2σ uncertainty of the final models is generally some 30–40 kg m^−3^ in even the lower crust (Supplementary Fig. 6). As such, the ∼90 kg m^−3^ difference that we find between any typical point beneath the suture zones and one in their surroundings is robust (*P*<0.01). This difference is even more profound if one considers the suture zones and the surrounding Plains as separate populations (Supplementary Fig. 8). On average, the YMS and CB lower crust (25–40 km) is 98 kg m^−3^ less dense than the Plains (the area east of 105.5° W) as a whole, and 87 kg m^−3^ less dense that the part of the Plains outside of the suture zones but in the same longitude range. The s.d. of the means of these groups are <2 kg m^−3^.

Thus although there are numerous sources of uncertainty with respect to the density at a discrete point in the model, the patterns in density structure and the magnitudes of the differences that we discuss are robust.

### Analytical stress modelling

Nature may abhor a vacuum, but all pressure variations are inherently at best metastable: If forces are to balance, disequilibria must be maintained (that is,, resisted) by flexural, viscous or other stress^58^. Our proof-of-concept analytical model is derived by idealizing the Proterozoic sutures as half of a 300-km wavelength, 2.5-dimensional cosine from 20 to 40 km depth with amplitude of −100 kg m^−3^ (Supplementary Fig. 11). We decompose this density model into its wavenumber-domain components and retain the dominant 8 terms of this Fourier expansion. Then the anomaly is separated into 1-km layers (centred at 20.5, 21.5, …, 39.5-km depth). Following ref. 59, we derive analytical solutions in a Newtonian rheology for each wavenumber-domain component and for each layer and sum their effects.

An elastic upper crust above this viscous material defines the necessary boundary conditions: no slip on the viscous-elastic interface. The horizontal shear stress at the base of the elastic material, which arises from the horizontal velocity gradient in the upper portion of the viscoelastic medium, requires horizontal normal stress in the elastic lid to maintain equilibrium. It is these stresses that are our primary interest.

A sinusoidal density perturbation of wavelength 2π/*k*, amplitude Δ*ρ*, and thickness *l* that is located a depth *d* below an elastic layer of thickness *h* imparts horizontal shear stress on their interface, which requires a vertically averaged horizontal normal stress in the overlying material to maintain force balance:


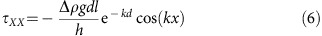


A positive value of 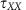 is defined as tensional in equation (6). Note that as long as the density anomaly resides in the top layer of a layered-viscosity medium, the stress imparted upon the material above is independent of the viscosity of the substrate^59^. Similar to Fig. 3b, there is ∼10 MPa of horizontal tension above the buoyant lower crust (Supplementary Fig. 11).

The same buoyant body exerts an upward vertical normal force (per unit area) on the overlying elastic crust, consistent with the diffuse rock uplift observed across the YMS and CB:


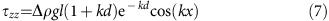


There are many other parameterizations that are possible, but no modification to the viscosity structure changes the polarity of the stress generated nor drastically affects its magnitude given the shallow, rather narrow anomaly with which we are concerned. Treating the entire system as uniformly elastic, viscoelastic or Newtonian all yield similar results: Buoyant lower crust encourages rock uplift and horizontal extension in the material above it.

### Finite-element model

The idealized structure used in the analytical solution shows unambiguously that buoyant material generates horizontal tension above it. To more accurately map the variations in stress due to the anomalous lower crust of the YMS and CB, however, it is important to use a more realistic structure. We use the mean density from across 1,000 models (Fig. 2) as the input for a three-dimensional PyLith finite-element model^60^. The mesh is 1,200 × 1,200 km and 300 km deep, divided into hexahedral elements of 10 × 10 km lateral dimensions. An elastic upper crust with 20-km thickness overlies Maxwell viscoelastic mantle. Dirichlet boundary conditions (that is,, no motion across boundary-planes, but free boundary-parallel slip) are applied on the sides and bottom of the volume. A uniform density is assigned to each cell: the average across the 1,000 density models. The uppermost layer (5 km elevation to sea level) is assigned a density of


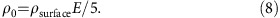


*E* is the average elevation within that cell, and *ρ*_surface_ is the average density from the Earth's surface to sea level in the final (that is,, average) density model. For example, a point at 1-km elevation with average density in the upper 1 km of 2,000 kg m^−3^ would be assigned a density of 400 kg m^−3^ in the finite-element model. Although this treatment of the density structure tricks PyLith into assigning unrealistic moduli to this layer, it exactly reproduces the lithostatic pressure at sea level. Below sea level, results are identical to meshes with topography explicitly included. All of the results that we discuss here and in the main text are referenced to depth below sea level. Variations in model geometry, rheology and duration are investigated in Supplementary Fig. 12 and are found to have negligible effects on our results. We also investigate the extent to which a regional stress field superposed with the gravity-derived stress may improve or degrade the fit to observations and find that gravity-derived stress appears dominant (Supplementary Fig. 13).

As is advocated in the PyLith 2.0.0 users' manual (ref. 61; page 155), an initial hydrostatic stress equal to average lithostatic pressure as a function of depth is applied in order to avoid large, spurious displacement when gravity is ‘turned on' 60. This initial condition mimics the theoretical framework of McGarr^58^, who argues that the stable state of stress in the absence of tectonic forcing is one of laterally uniform, isotropic stress everywhere equal to lithostatic pressure (again, the weight per unit area of the overburden). Departures from this state (that is,, the difference between the initial condition applied and the stress field after gravity is ‘turned on' at the beginning of a PyLith simulation) create deviatoric stress.

The 2D model shown in Fig. 3C is identical except that the elements are 5 × 5 km in the elastic medium (5 km elevation to 15 km below sea level) and 10x10 km below. The density assigned to each element is the average of the final density model (that is,, mean across our 1,000 simulations) projected from 100 km either side of fence diagram A-A'.

### Time- and viscosity-independence of modelled stress

Our overarching aim is to improve the understanding of and preparedness for intraplate earthquakes in the central United States. Therefore, we are concerned with the modern state of stress and not with the future evolution of the North American lithosphere. Thus our approach differs from the bulk of geodynamics literature, which generally develops time-transgressive models to explain the causes of modern processes and origins of modern structures. Nevertheless, we acknowledge that strain in viscoelastic media and therefore the spatial pattern of stress generated in surrounding material is an inherently time-dependent process.

To explore how sensitive the modelled loading of the elastic crust is to the time since density variations in the viscoelastic material were introduced and strain began to accrue (that is,, ‘where we are' on the geodynamic timeline of the region), we run a series of synthetic tests. Specifically, we compare the stress in the seismogenic crust shortly after the simulation initiated and at times as great as 50 Myr later for viscosities of 10^22^–10^24^ Pa s for example, ref. 30. For computational ease, these consisted of 2D finite-element models with a relatively simple structure: a −75 kg m^−3^ density anomaly from 20 to 40 km depth and 100 km wide in an otherwise uniform Maxwell viscoelastic medium overlain by elastic crust. Elements were 10-km scale triangles; mesh extended 1,000 km laterally and 300 km vertically.

We find that a body of these dimensions is relatively stable over geologic time; maximum total strains in the viscoelastic lower crust were of the order of 10^−5^ after 50 million years . More important for the present purpose, we find that the time- and viscosity-dependence of differential stress in the elastic crust is negligible. The modelled stress after the first time step (that is,, a quasi-static simulation) was nearly identical to the modelled stress after tens on Myr and at different viscosities within the range we explored (Supplementary Fig. 12). Therefore we conclude that our approach—using a quasi-static simulation and poorly constrained viscosity of 10^23^ Pa s—yields robust estimates of the spatial pattern and magnitude of modern deviatoric stress in the seismogenic crust. Said differently, it does not matter if the modern density structure came to be 60 million years ago or 60, the modern density structure allows a realistic appraisal of the modern stress. Obviously other time-dependent processes, especially sedimentation/erosion, will change the state of stress may create positive feedback between deformation and surface processes. While these processes are undoubtedly important for the geodynamic evolution of the region, they contribute to the modern stress field only primarily insofar as they have helped to shape the modern density structure (time-dependent stress perturbations from postglacial rebound or erosion are predicted to have magnitudes of <1 MPa for example, ref. 62).

### Topographic profile

The topographic profile of the Nussbaum surface (Fig. 3d,e) was created by first digitizing a 100 km long profile line using Esri's ArcGIS software. The line was then converted to a series of points with 30 m spacing. Elevation values were assigned to each point by extracting height above sea level (m) from a USGS National Elevation Dataset (NED) 30 m grid cell resolution digital elevation model.

### Earthquake catalogue and moment tensors

The base of our earthquake catalogue is the National Seismic Hazard Mapping Project declustered catalogue^39^. All events greater than estimated *M*_w_4.0 are retained, as are more recent (since 1980) events of *M*_w_3.0 or greater. Finally, we remove events spatiotemporally associated with wastewater injection^39^, but we do display moment tensor principal stress axes from induced events. Moment tensors were taken from ‘earthquakes.usgs.gov/search'. For clarity, not all ∼350 moment tensors in northcentral Oklahoma and southcentral Kansas are included. In this area, we plot optimal horizontal principal tension directions from stress inversions of spatial groups of moment tensors.

### Data availability

Seismic velocity models were provided by Dr Weisen Shen. Other inputs, the density and stress models, density modelling code and PyLith .cfg files are available from W.L.: wlevandowski@usgs.gov. Please address all enquiries to W.L.

## Additional information

**How to cite this article:** Levandowski, W. *et al*. Gravitational body forces focus North American intraplate earthquakes. *Nat. Commun.* **8,** 14314 doi: 10.1038/ncomms14314 (2017).

**Publisher's note**: Springer Nature remains neutral with regard to jurisdictional claims in published maps and institutional affiliations.

## Supplementary Material

Supplementary Information Supplementary Figures and Supplementary References
