# Supplementary material for: Gravitational body forces focus North American intraplate earthquakes
Source: Nat Commun. 2017 Feb 17;8:14314. doi: 10.1038/ncomms14314 (PMC5321685; doi:10.1038/ncomms14314)
Supplement: Supplementary Information — Supplementary Figures and Supplementary References [file ncomms14314-s1.pdf]

Supplementary Figure 1: **Gravity and Topography data and models**. (**A**) Observed elevation, smoothed to account for lithospheric flexural strength. (**B**) Observed free air gravity variations. (**C**) Predicted elevation from the mean of the initial, seismically derived estimates of density (shown in Supplementary Figure 2), smoothed to account for lithospheric flexural strength. (**D**) Free air gravity variations from the mean of the initial models. (**E-F**) Topography and gravity residual from the mean of the initial model, defined as prediction minus observation. Cool colors connote regions in which the density is underestimated. On the YMS and CB, gravity is predicted to be greater than observed. By contrast, the elevation of the southwestern part of the YMS is modestly less than predicted. (**G-H**) Residuals remaining after the densities have been refined (i.e., to the values shown in Fig. 3 of the main text). Remaining residuals are universally less than 5 mGal (L1 norm 2.1 mGal) and 50 meters (L1 norm, 24 meters).

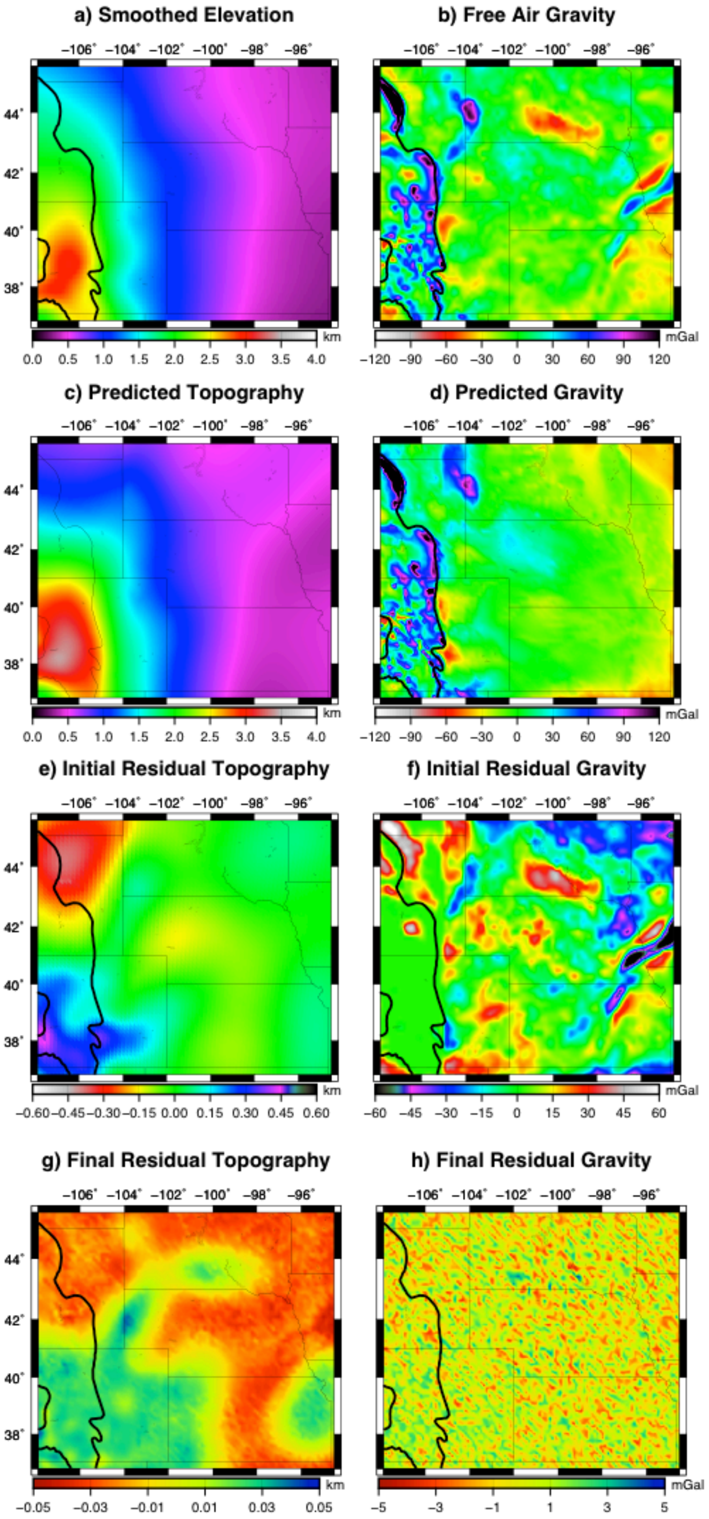

Supplementary Figure 2: **Initial density estimates** based on seismic velocity. The lack of Mesozoic and Cenozoic sediments on the YMS and CB is manifest in the high densities in (A) and contrasts strongly with the Powder River basin north of the CB, the Denver-Julesburg basin between the two suture zones, and the Anadarko basin southeast of the YMS. By contrast, (D) shows that the lower crust beneath the YMS and CB is some 50 kg/m^3 less than beneath the surrounding Great Plains.

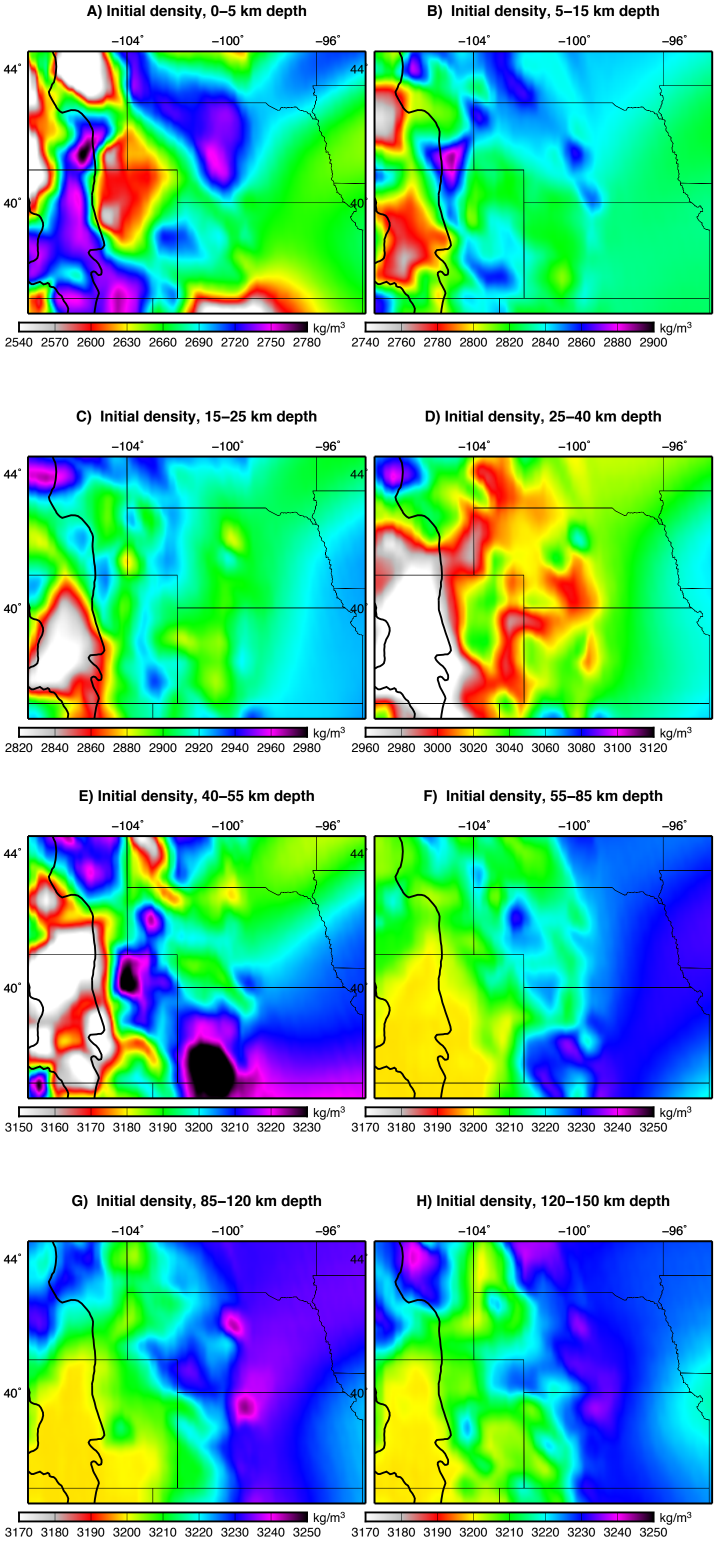

Supplementary Figure 3: **Adjustments to the initial density model** that are required to reproduce gravity and topography. Most of these adjustments are small in magnitude compared to the lateral variations estimated from seismic velocity at any given depth

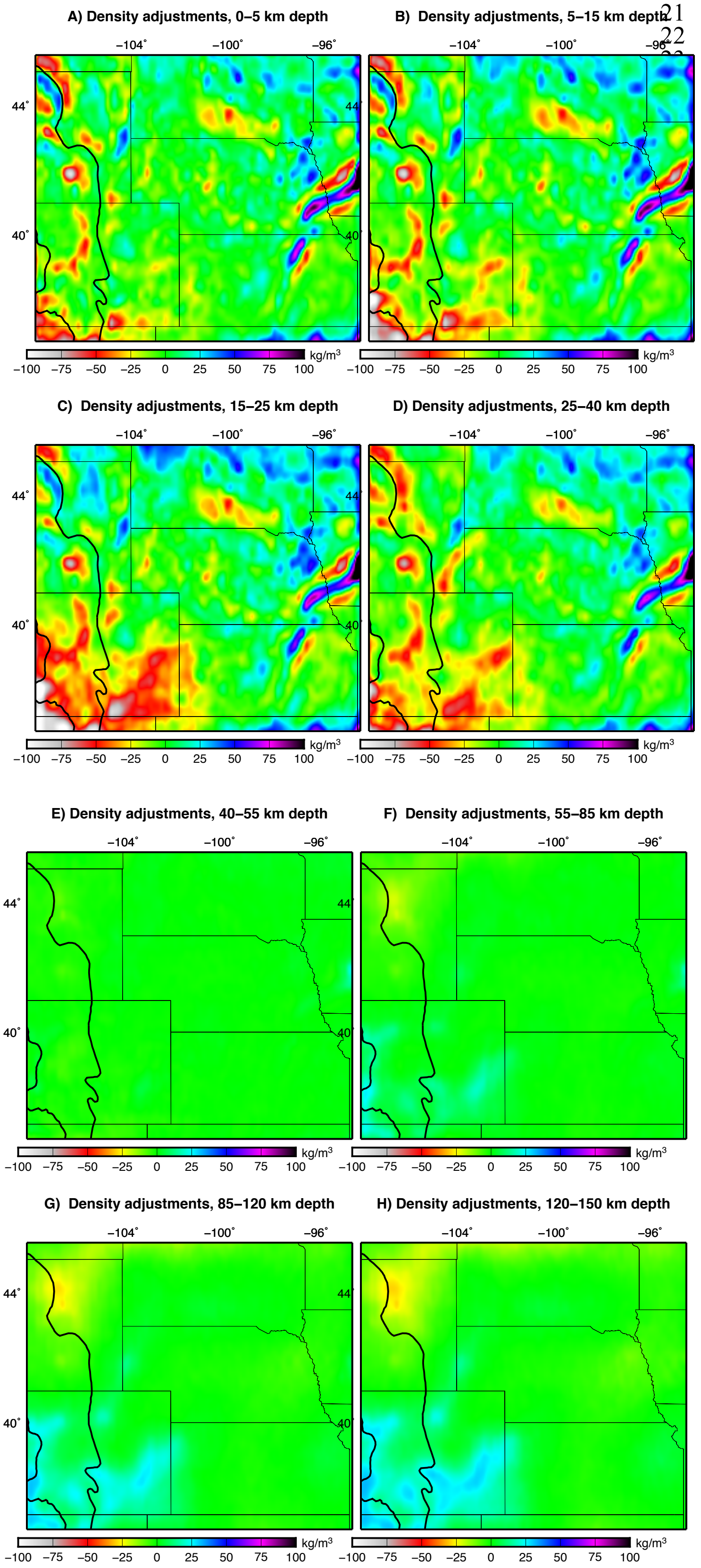

(generally <30 kg/m^3 compared to ±100 kg/m^3), small in horizontal extent (below the ~100-km resolution of the Transportable Array surface wave data), or both.

Supplementary Figure 4: **Uncertainties in the initial density models**. This uncertainty results directly from the variety of 1-D velocity models (the posterior distributions of Shen et al., shown here at the Transportable Array seismometers at which they are derived) that are capable of jointly reproducing surface wave dispersion curves and receiver functions. Uncertainties are highest at points in the upper crust and near the Moho (e.g., D-F), where—depending on the 1-D profile chosen—a specific depth-point may be variably in the crust or in the mantle.

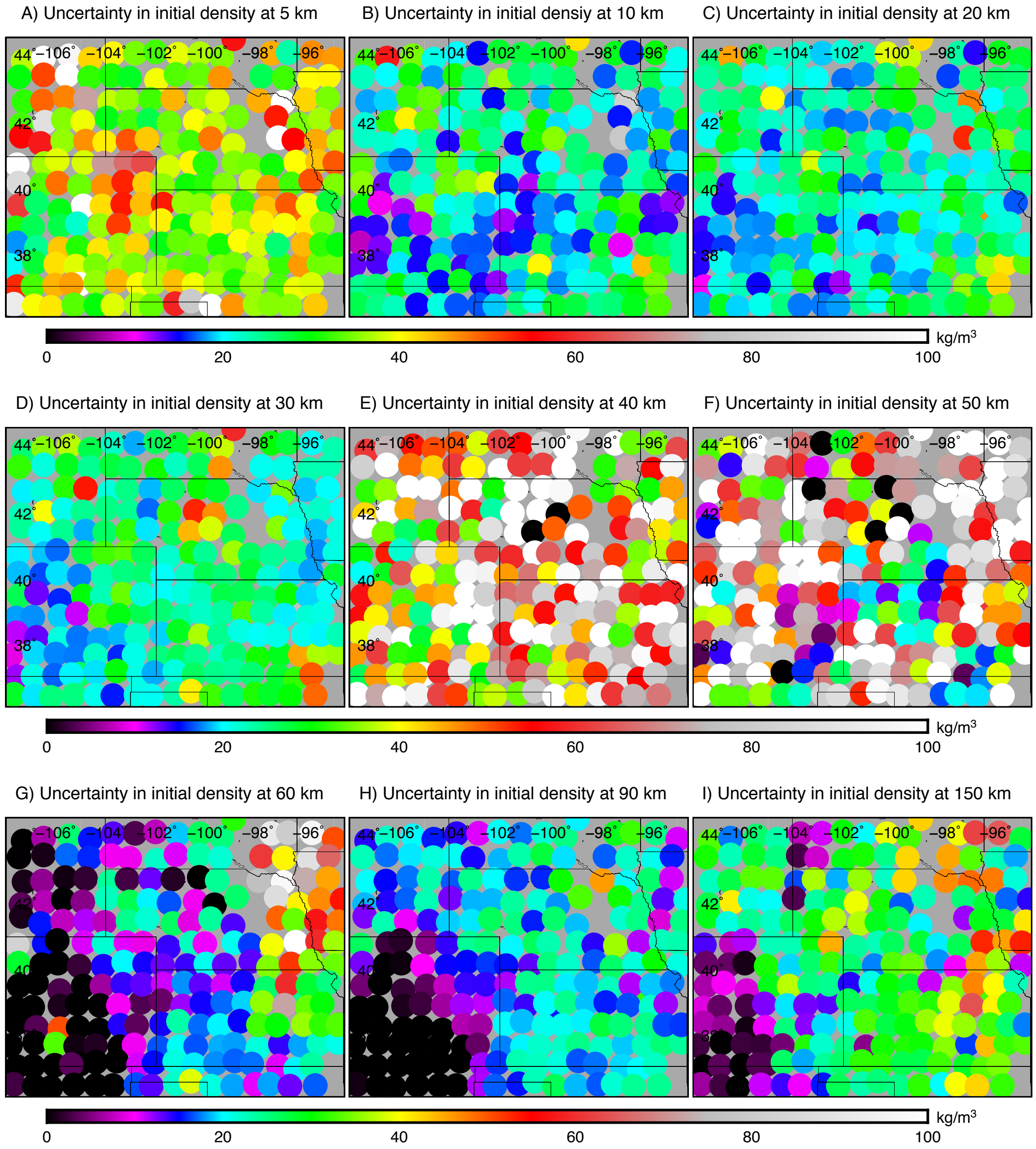

Supplementary Figure 5: **1-D profiles of average the 95% confidence intervals of initial estimates** of velocities (Left) and attendant densities (Right) at 1-km intervals beneath each station. Blue dots are the uncertainties averaged across all stations; magenta dots show only the averaged uncertainties of stations within the YMS or CB. Results are quite similar for both sets of stations. Uncertainty is highest in the upper crust and near the Moho. Thus, not only is a wide range of density plausible for any given velocity (see section S2.4.2) but also a range of velocities—and densities, as inferred simply from eq. S7-8—at a given point can reproduce surface wave dispersion curves and receiver functions. Thus, the adjustments made to the initial density models (estimated from 1-D velocity/crustal thickness models and eqs. S7-8), which are generally <40 kg/m^3, are not only reasonable given the fact that a range in density of ~±150 kg/m^3 is observed in rocks of nearly identical velocities (see Supplementary sections S2 and S3, below) but also are within the range of densities estimated solely from seismic velocity. We also show the uncertainty of the 5-km moving average of velocity/density beneath each station (thin lines). As seen in the right panel, even when averaged over the thickness of one of the cells used in our inversion, the uncertainty of the initial density models in the lower crust beneath a given station is some 120 kg/m^3, much greater in magnitude than the adjustments made to the density structure (for example, 50 kg/m^3 in the YMS and 30 kg/m^3 in the CB; Supplementary Figure 3D). The final density models are within the 95% bounds of the initial distribution, and indeed less than 1 standard deviation from the mean thereof in the YMS and less than one half of one standard deviation from the mean thereof in the CB.

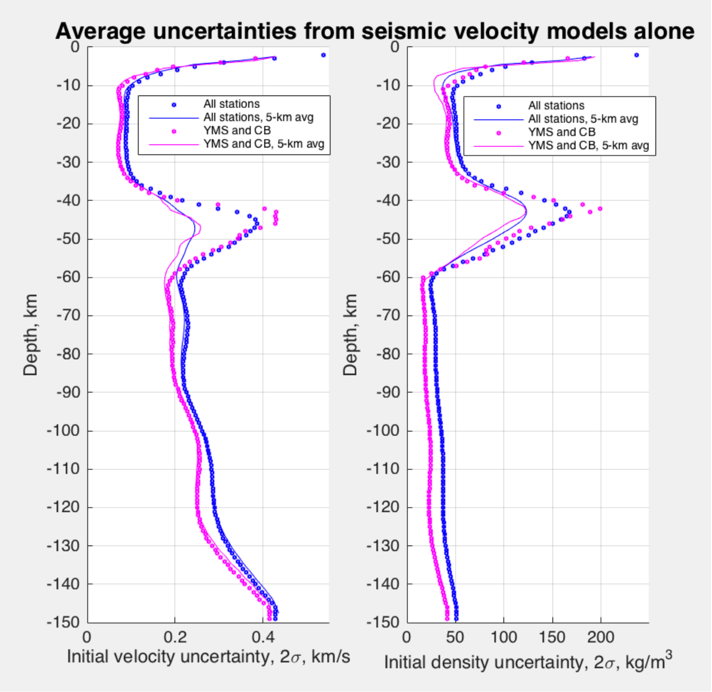

Supplementary Figure 6: **Uncertainty of the final models** (2σ, or the 95% confidence range of the posterior distribution) in the depth ranges indicated. Of particular importance to this study is the lower crust (D-E). The ~90 kg/m^3 difference between the YMS/CB and the rest of the region is robust: typical 1σ uncertainty in the 30-40 km depth range beneath a given point is 14 kg/m^3, so uncertainty of the difference between two such points is $(14^2+14^2)^{0.5} \approx 20$ kg/m^3, and the 90 kg/m^3 difference is significant to >4σ (p<0.0001).

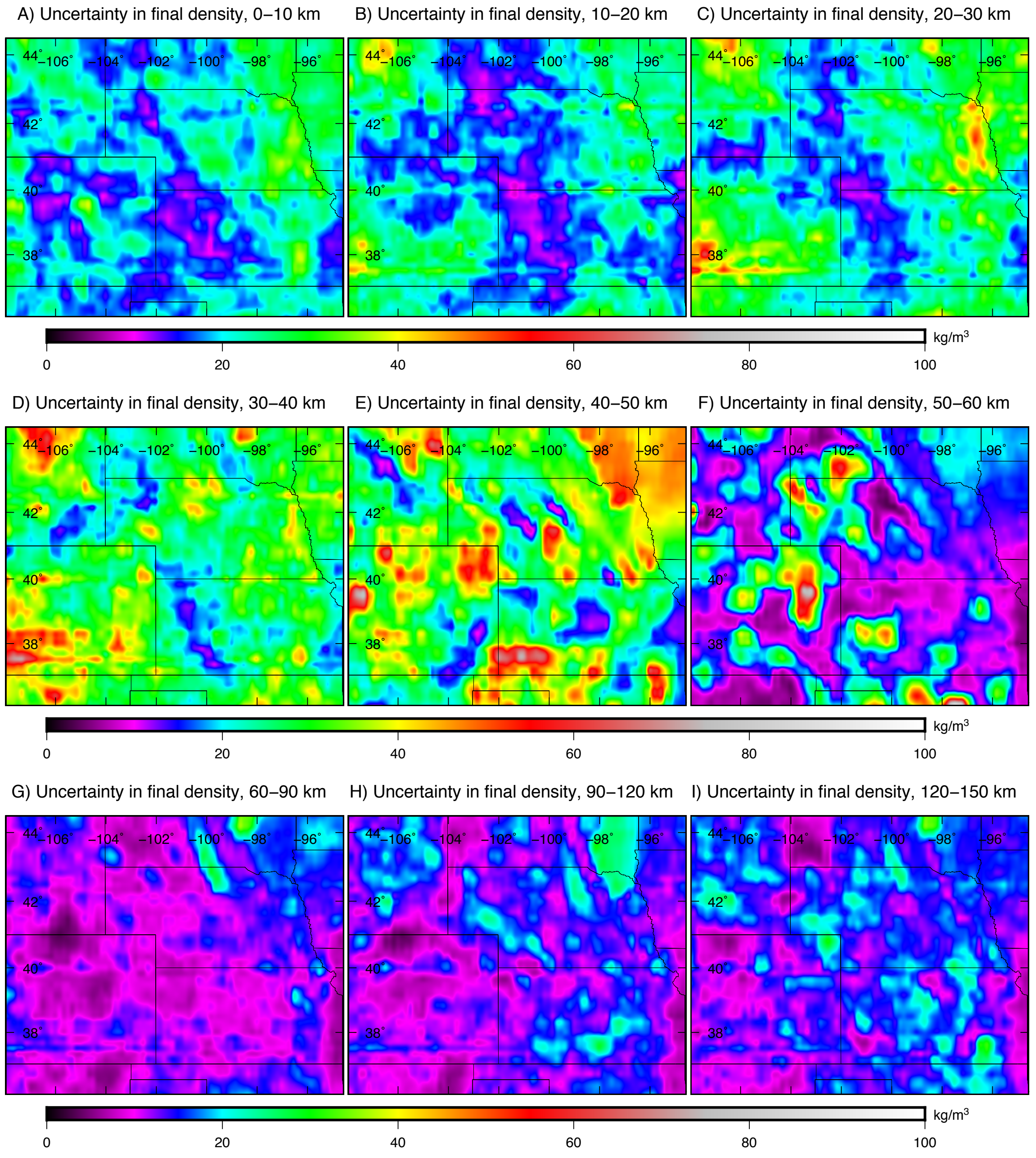

Supplementary Figure 7: **Scatterplots of the initial and final uncertainties** averaged over the depth ranges specified. Gray line is 1:1. The final uncertainty is largely controlled by the initial uncertainty (i.e., the variability of the seismic velocity models) at all depths. The best correlation and the greatest overall uncertainties are in the lower crust (middle row). The overall correlation is to be expected, since the lateral variations in initial density at any given depth (e.g., Supplementary Figure 2) are >100 kg/m^3, but the adjustments are generally some very few tens of kg/m^3 (e.g., Supplementary Figure 3). In other words, the initial models dominate the final result, and they also dominate the uncertainty thereof. Such a finding underscores the benefit of using not one velocity model but as many models as are available that reproduce the chosen seismic data.

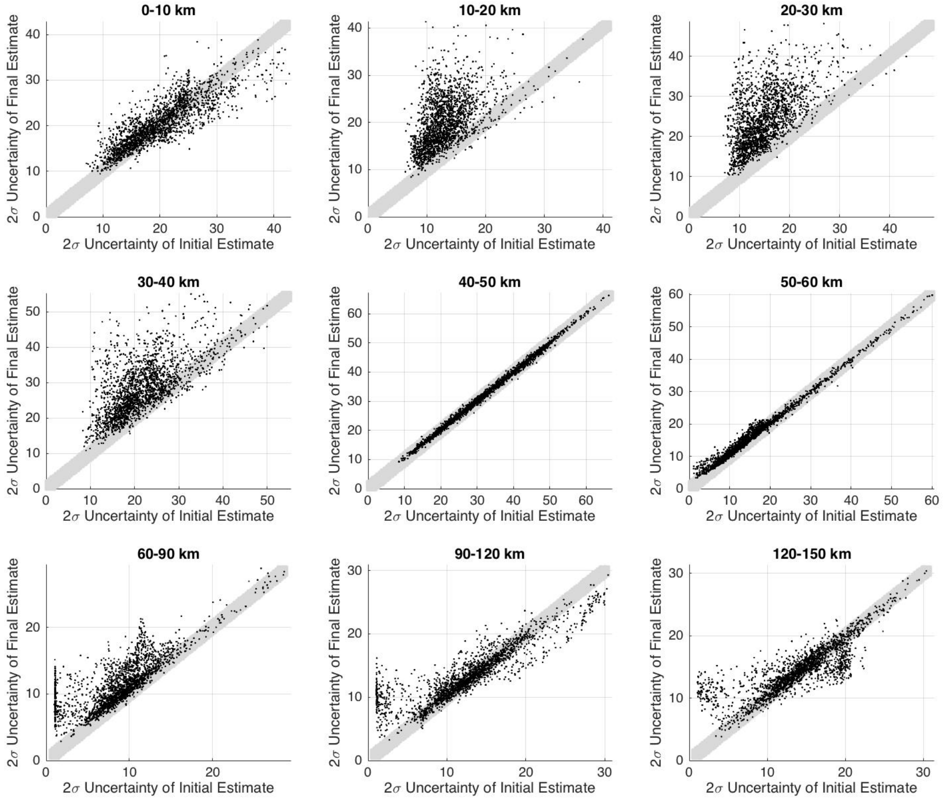

Supplementary Figure 8: **Histograms of the mean final density in the lower crust** beneath the YMS and CB (red), beneath the entire study area outside of the sutures and east of 105.5°W (blue) and the areas outside of the sutures and at similar longitudes (105.5–101W, green).

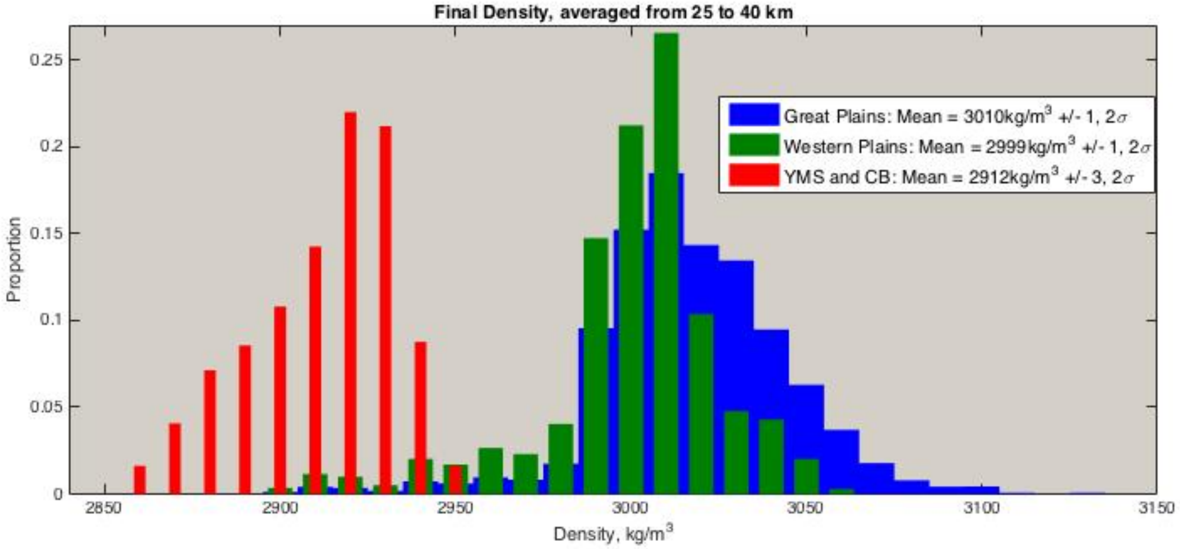

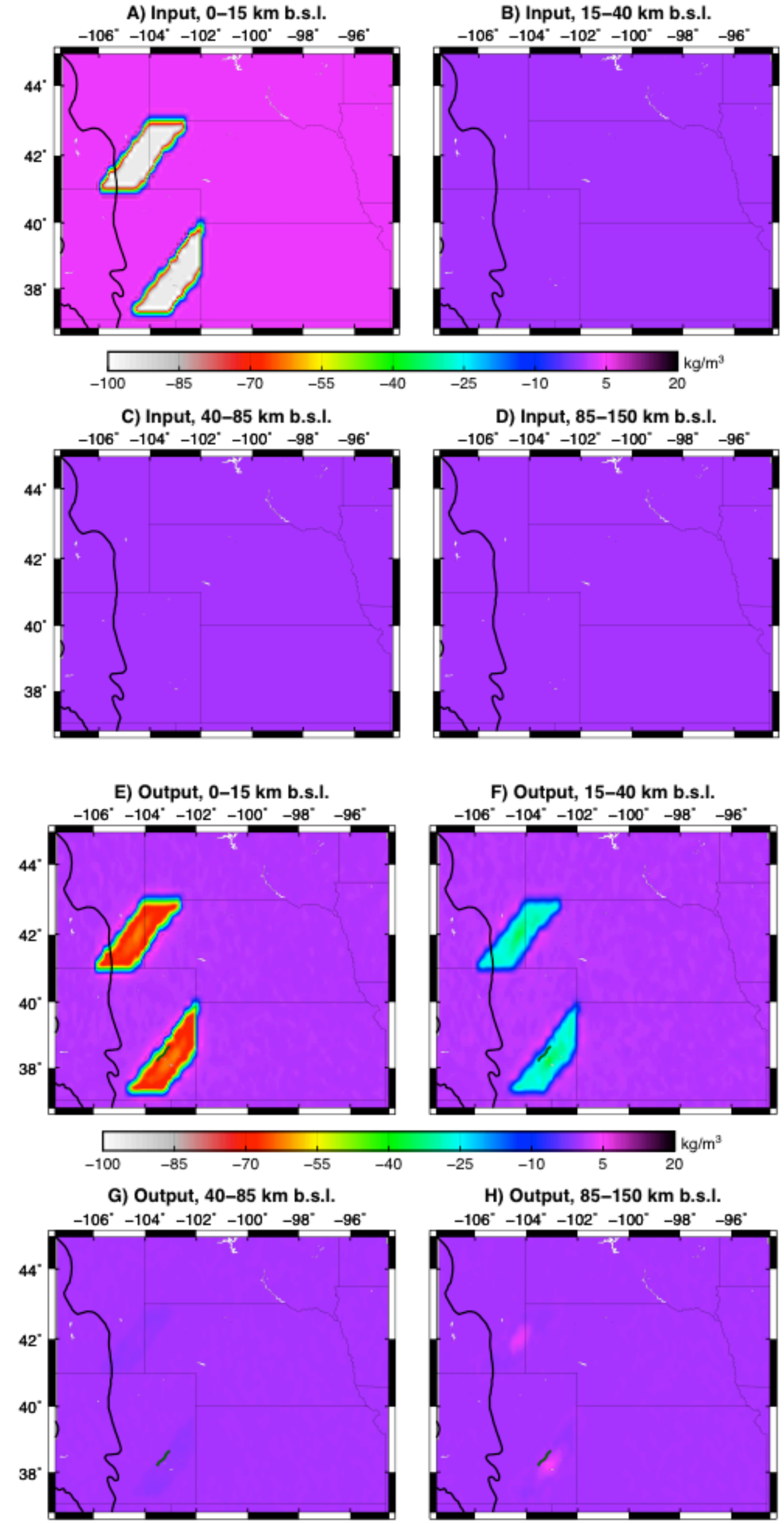

Supplementary Figure 9: **Feature recovery test for buoyant upper crust**. The input -100 kg/m^3 density anomaly from 0-5 and 5-15 km depth is modeled as -70 kg/m^3 from 0-5 and 5-15 km and -25 kg/m^3 from 15-25 and 25-40 km.
The buoyant lower crust beneath the CB and YMS is unlikely to be an artifact due to material above.

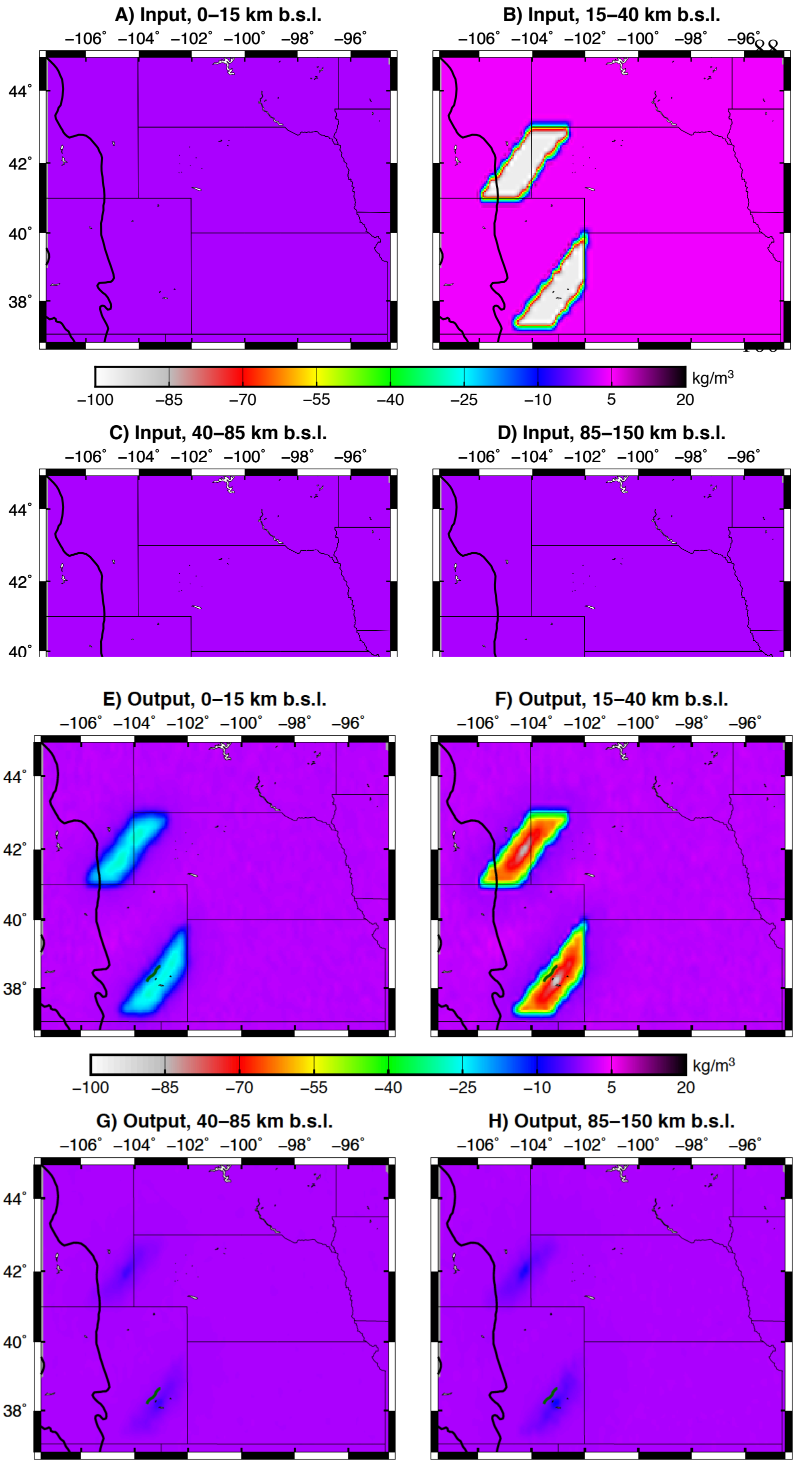

Supplementary Figure 10: **Feature recovery test for buoyant lower crust**. The input -100 kg/m^3 density anomaly from 15-25 and 25-40 km depth is modeled as -25 kg/m^3 from 0-5 and 5-15 km and -80 kg/m^3 from 15-25 and 25-40 km.
Therefore, the magnitude of the anomaly discussed in the main text is likely to be a conservative estimate.

Supplementary Figure 11:

Analytical model of gravity-derived stress. Buoyant lower crust generates horizontal tension in the material above it. The solid blue line is the average horizontal tension (tension is positive in this figure) through a 20-km elastic lid overlying a 20-km thick body with uniform density (shown in red). The vertical normal stress (positive upward) at the interface between the two media is shown by the dashed line. In an elastic medium, the upward vertical normal stress is resisted by flexural strength, leading to vertical compressive stress (and augmenting differential horizontal tension). In a fluid, the basal normal force would cause uplift; this increased elevation would create a hydrostatic pressure contrast and similarly increase deviatoric tension.

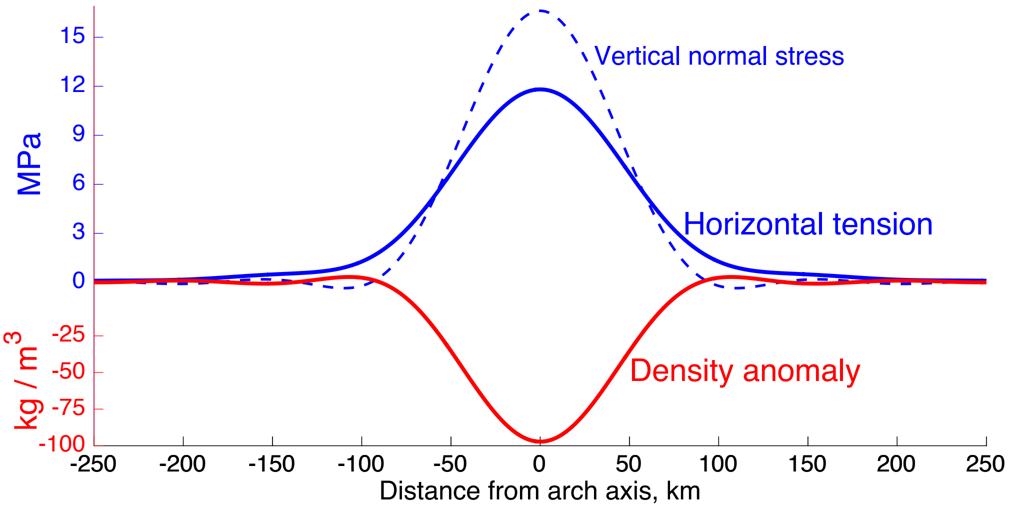

Supplementary Figure 12:

Time- and viscosity-independence of stress in the brittle crust Time-slices through the synthetic deformation model (visualized with ParaView), with only the elastic crust shown. Tension defined as negative. A 100 km-wide body with density anomaly of -75 kg/m^3 sits from 20-40 km depth beneath this elastic layer. The rest of the underlying viscoelastic substrate is uniform. The stress field in the elastic crust does not evolve appreciably over time, nor does it depend strongly on viscosity.

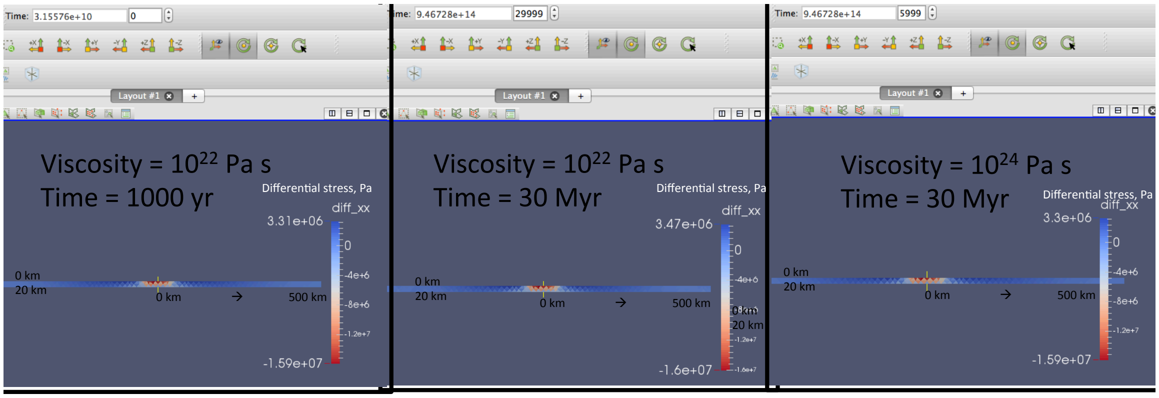

Supplementary Figure 13: **Gravity-derived stress tensor plus far-field stress** Black arrows are observed minimum horizontal compression direction. Gray arrows are predictions. Colorscale depicts deviatoric stress

a) Our model: same as Fig. 3a in the main text.

b) The modeled stress tensors in (a) plus 1 MPa of uniaxial horizontal compression oriented N45E. The fit to observations is similar to (a): slightly better in the Raton basin, slightly worse in the Denver basin, northern Nebraska, and along the Midcontinent Rift.

c) Our model plus 2.5 MPa of uniaxial horizontal compression oriented N45E. Fit is markedly worse than (a) everywhere except the YMS and CB.

d) Our model plus 5 MPa of uniaxial horizontal compression oriented N45E. Because the far-field stress now dominates the net stress tensor, the observed variability in focal mechanisms is not reproduced.

e) Our model plus 2 MPa of uniaxial horizontal compression oriented north-south. Fit improves (relative to gravity-derived stress alone) in the Denver basin and northern Nebraska but deteriorates (relative to gravity-derived stress alone) in the Raton basin, southern Kansas, the YMS, and the CB.

f) Our model plus 2 MPa of uniaxial horizontal compression oriented N15E. Fit deteriorates everywhere except the Midcontinent Rift and northern Nebraska.

g) Our model plus 2 MPa of uniaxial horizontal compression oriented N30E. Fit does not markedly improve anywhere and deteriorates in the Denver and Raton basins.

h) Our model plus 2 MPa of uniaxial horizontal compression oriented N60E. Fit is generally similar to our model, with a slight improvement in the Denver basin and deterioration in southern Kansas and the southwestern YMS.

i) Our model plus 2 MPa of uniaxial horizontal compression oriented N75E. Fit improves slightly in the Midcontinent Rift, northern Kansas, and the Denver basin but deteriorates substantially in southern Kansas and the Raton basin.

j) Our model plus 2 MPa of uniaxial horizontal compression oriented east-west. Fit degrades in the Denver and Raton basins and along the YMS and CB but improves somewhat in southern Kansas, the Midcontinent Rift, and northern Nebraska.

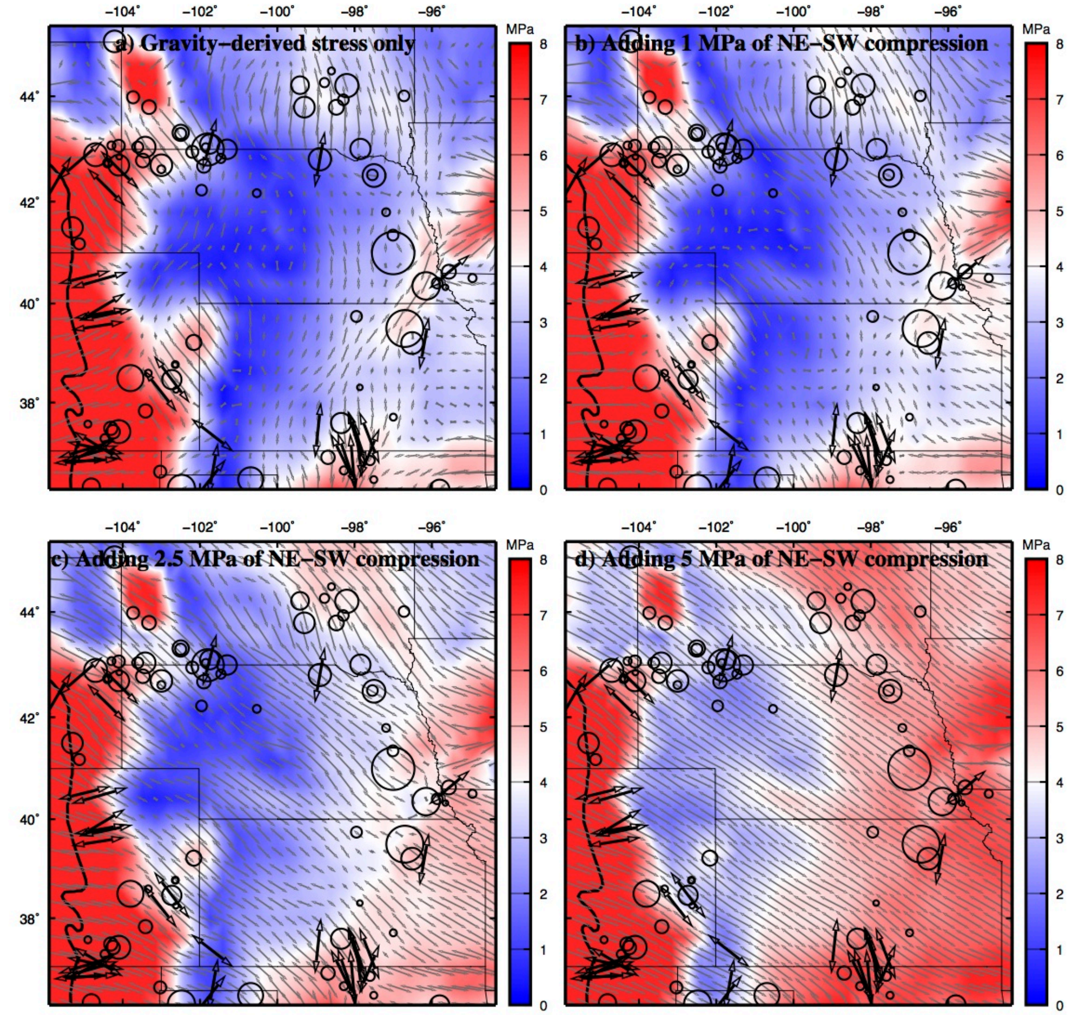
a) Gravity−derived stress only
b) Adding 1 MPa of NE−SW compression
c) Adding 2.5 MPa of NE−SW compression
d) Adding 5 MPa of NE−SW compression
MPa
−104˚
−102˚
−100˚
−98˚
−96˚
44˚
42˚
40˚
38˚
0
1
2
3
4
5
6
7
8

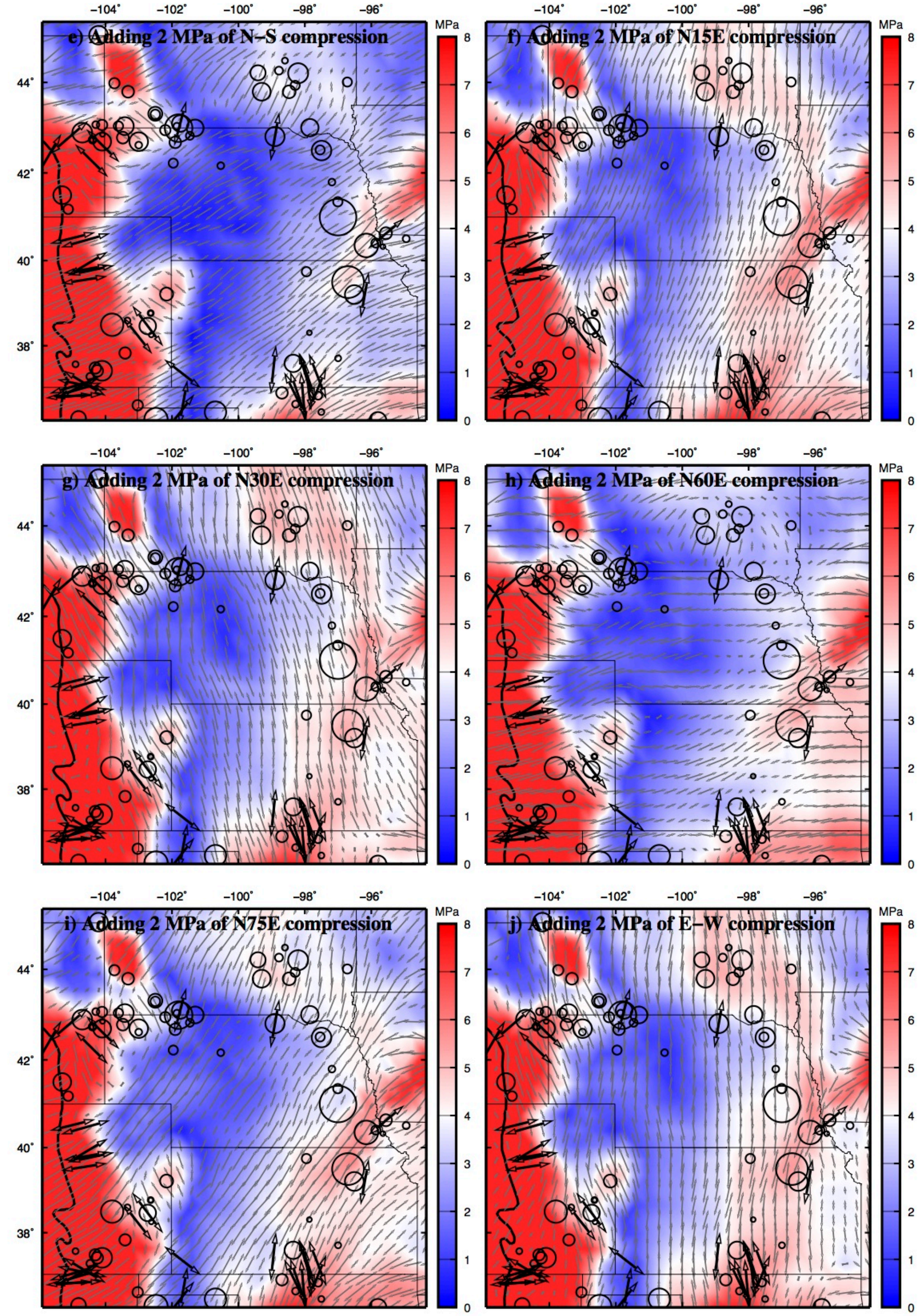
e) Adding 2 MPa of N–S compression
f) Adding 2 MPa of N15E compression
g) Adding 2 MPa of N30E compression
h) Adding 2 MPa of N60E compression
i) Adding 2 MPa of N75E compression
j) Adding 2 MPa of E–W compression
−104˚
−102˚
−100˚
−98˚
−96˚
44˚
42˚
40˚
38˚
MPa
0
1
2
3
4
5
6
7
8

Supplementary References:

1. Levandowski, W., Boyd, O., Briggs, R. & Gold, R. A random-walk algorithm for modeling lithospheric density and the role of body forces in the evolution of the Midcontinent Rift. *G3* (2015). doi:10.1002/2015GC005961
2. Christensen, N. I. Serpentinites, peridotites, and seismology. *Int. Geol Rev.* **46,** 795–816 (2004).
3. Jones, C. H., Mahan, K. H., Butcher, L. A., Levandowski, W. B. & Farmer, G. L. Continental uplift through crustal hydration. *Geol* **43,** 355–358 (2015).
4. Lee, C.-T. A. Compositional variation of density and seismic velocities in natural peridotites at STP conditions: Implications for seismic imaging of compositional heterogeneities in the upper mantle. *JGR* **108,** 2441 (2003).
5. Schutt, D. L. & Lesher, C. E. Compositional trends among Kaapvaal Craton garnet peridotite xenoliths and their effects on seismic velocity and density. *EPSL* **300,** 367–373 (2010).
6. Keller, G. R. & Stephenson, R. A. in *4-D Framework of Continental Crust* **200,** 127–143 (Geological Society of America, 2007).
7. Walcott, R. I. Flexural rigidity, thickness, and viscosity of the lithosphere. *JGR* **75,** 3941–3954 (1970).
8. Calais, E., Freed, A. M., Van Arsdale, R. & Stein, S. Triggering of New Madrid seismicity by late-Pleistocene erosion. *Nature* **466,** 608–611 (2010).
9. Ghosh, A. & Holt, W. E. Plate Motions and Stresses from Global Dynamic Models. *Science* **335,** 838–843 (2012).
10. Ghosh, A., Holt, W. E. & Wen, L. Predicting the lithospheric stress field and plate motions by joint modeling of lithosphere and mantle dynamics. *JGR* **118,** 346–368 (2013).
11. Forte, A. M., Moucha, R., Simmons, N. A., Grand, S. P. & Mitrovica, J. X. Deep-mantle contributions to the surface dynamics of the North American continent. *Tectonophysics* **481,** 3–15 (2010).
12. Forte, A. M., Mitrovica, J. X., Moucha, R., Simmons, N. A. & Grand, S. P. Descent of the ancient Farallon slab drives localized mantle flow below the New Madrid seismic zone. *GRL* **34,** L04308 (2007).
